# Near-infrared fluorescence image-guidance in plastic surgery: A systematic review

**DOI:** 10.1007/s00238-018-1404-5

**Published:** 2018-02-27

**Authors:** Anouk J. M. Cornelissen, Tom J. M. van Mulken, Caitlin Graupner, Shan S. Qiu, Xavier H. A. Keuter, René R. W. J. van der Hulst, Rutger M. Schols

**Affiliations:** 1 Department of Plastic, Reconstructive and Hand Surgery, Maastricht University Medical Center, P. Debyelaan 25, 6229 HX Maastricht, The Netherlands; 2 NUTRIM School of Nutrition and Translational Research in Metabolism, Maastricht University, Maastricht, The Netherlands

**Keywords:** Plastic surgery, Reconstructive surgery, Microsurgery, Near-infrared fluorescence imaging, Anatomical navigation, Tissue perfusion assessment, Image-guided surgery

## Abstract

**Background:**

Near-infrared fluorescence (NIRF) imaging technique, after administration of contrast agents with fluorescent characteristics in the near-infrared (700–900 nm) range, is considered to possess great potential for the future of plastic surgery, given its capacity for perioperative, real-time anatomical guidance and identification. This study aimed to provide a comprehensive literature review concerning current and potential future applications of NIRF imaging in plastic surgery, thereby guiding future research.

**Methods:**

A systematic literature search was performed in databases of Cochrane Library CENTRAL, MEDLINE, and EMBASE (last search Oct 2017) regarding NIRF imaging in plastic surgery. Identified articles were screened and checked for eligibility by two authors independently.

**Results:**

Forty-eight selected studies included 1166 animal/human subjects in total. NIRF imaging was described for a variety of (pre)clinical applications in plastic surgery. Thirty-two articles used NIRF angiography, i.e., vascular imaging after intravenous dye administration. Ten articles reported on NIRF lymphography after subcutaneous dye administration. Although currently most applied, general protocols for dosage and timing of dye administration for NIRF angiography and lymphography are still lacking. Three articles applied NIRF to detect nerve injury, and another three studies described other novel applications in plastic surgery.

**Conclusions:**

Future standard implementation of novel intraoperative optical techniques, such as NIRF imaging, could significantly contribute to perioperative anatomy guidance and facilitate critical decision-making in plastic surgical procedures. Further investigation (i.e., large multicenter randomized controlled trials) is mandatory to establish the true value of this innovative surgical imaging technique in standard clinical practice and to aid in forming consensus on protocols for general use.

Level of Evidence: Not ratable

## Introduction

Innovative optical imaging methods can be applied during surgery to detect and to differentiate tissues [1], a technique also known as image-guided surgery. A promising modality is near-infrared fluorescence (NIRF) imaging. After administration, contrast agents with fluorescent characteristics (i.e., fluorophores or fluorescent dyes) in the near-infrared range (NIR 700–900 nm) can be visualized using dedicated NIR camera systems. These fluorophores can be injected systemically (e.g., intravenously) or locally (e.g., subcutaneously). Indocyanine green (ICG) is the most common dye [2], but a variety of fluorophores can be applied. Currently, novel dyes with different chemical properties are being developed or tested in a preclinical setting in order to expand the potential of tissue differentiation, nerve detection in particular. Arteries, veins, ureters, lymph vessels, and lymph nodes have already successfully been identified using NIRF imaging in clinical trials [3–5]. A uniform approach regarding timing, dosage, and route of dye administration has not yet been established. The optimization of both imaging systems and fluorescent dyes is essential to improve current shortcomings [3].

A NIRF imaging system can be used by the surgeon in real time, thereby providing a significant advantage in terms of perioperative anatomical navigation and identification as well as facilitating the assessment of tissue perfusion or viability [1]. The NIRF imaging technique is currently being implemented in most new microscopic surgical systems. Since many plastic surgery departments possess a microscope, it will probably become easily accessible for the general field.

This review aims to provide a comprehensive insight into the current and potential future applications of NIRF imaging for perioperative anatomical guidance in the field of plastic and reconstructive surgery. Directions and implications for future research are given.

## Methods

This study was conducted according to the PRISMA standard for systematic reviews (see *Electronic Supplementary Material* for PRISMA Checklist) [6]. A systematic literature search was performed in October 2017 in the following databases: Cochrane Library database CENTRAL, MEDLINE, and EMBASE. Both structured MeSH terms and free terms were used in the PubMed search. The terms applied were such that any description that could resemble or relate to the use of NIRF imaging in plastic and reconstructive surgery would be uncovered by the search; Table 1 displays an overview of the search terms. Additional literature was collected after scanning the reference lists of existing review articles.

**Table 1:** An overview of search terms

MESH	Free
Plastic surgery Microsurgery Reconstructive surgical procedures	Plastic surgery Microsurgery Reconstructive surgery Reconstructive surgical procedure
Near-infrared fluorescence imaging Optical imaging	Near-infrared fluorescence imaging Near-infrared fluorescence Near-infrared Fluorescence imaging Optical imaging

Two investigators (R.S. and A.C.) independently performed the literature selection. A third investigator (X.K.) was available for consultation in case of disagreement. Inclusion of an article resulted from a three-phase process that consisted of the initial literature search, screening of the literature resulting from the search, and evaluation of eligibility of the articles provided by the screening. Neither language nor publication date or publication status restrictions were applied. Both clinical and preclinical studies were included; systematic reviews and meta-analysis were excluded. A substantive evaluation of NIRF systems and their corresponding NIRF imaging performance is not within the scope of this review.

Eligibility of the studies was based on the following criteria:

- Does the study report on NIRF imaging in plastic and reconstructive surgery?
- Does the paper describe an application of NIRF imaging for enhanced anatomical guidance or assessment of tissue perfusion?
- Does the article provide insight into future applications of NIRF image-guided plastic surgery?

Primarily, titles and abstracts were screened. In case of incertitude, full-text reports were read to determine eligibility. Reference lists of the selected articles were also screened based on the previously described criteria. A data extraction sheet was developed containing items on the aim of the study, the imaging system that was used, and the fluorescent dye and administration. The data extraction sheet was completed for all eligible studies by three independent authors (A.C., R.S., and C.G.).

## Results

**Fig. 1:**
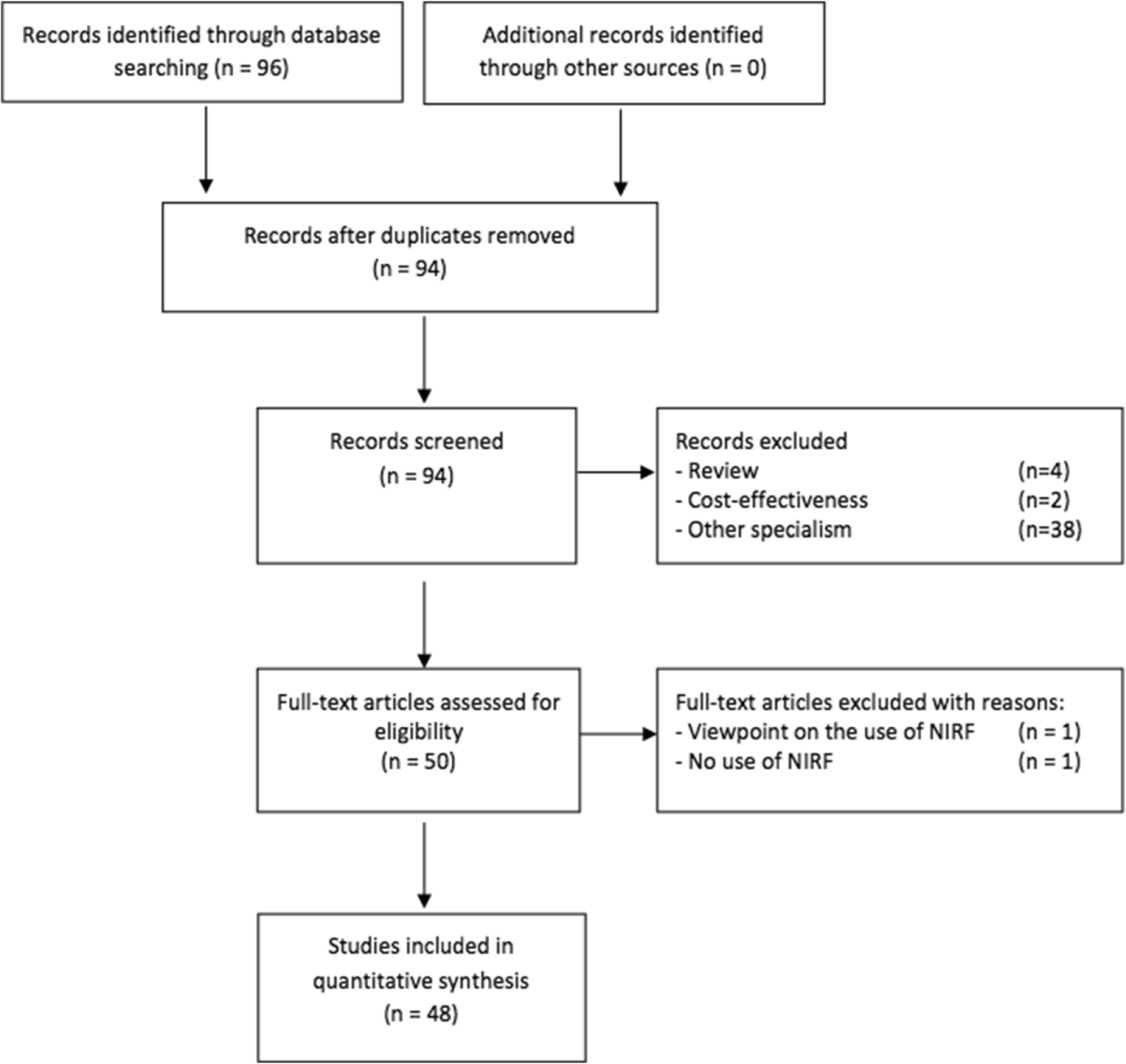
Flow diagram of the literature search according to PRISMA statement

Following the systematic literature search, a total of 94 studies were identified. After reviewing the title and abstract, 44 hits were directly excluded. Another two were excluded after reading the full article. The main reason for exclusion: NIRF imaging was used in another surgical specialty than plastic and reconstructive surgery (*n* = 38, e.g., general surgery, neurosurgery, urology, or dermatology). Other reasons for exclusion: NIRF was used in a molecular study, a review was presented (*n* = 4), or the cost-effectiveness of the device itself was explored (*n* = 2). A detailed overview of the study selection is presented in Fig. 1.

Ultimately, 48 studies were eligible within the scope of this review (covering a total of 1166 animal/human subjects). The selected studies—all written in the English language—were published within the period from 2007 until 2017. Fifteen articles reported on animal experiments, and the remainder described clinical findings. The content of the selected articles will be presented following three subcategories, respectively: NIRF imaging systems (see Table 2), NIR fluorescent dyes (see Table 3), and applications of NIRF imaging in plastic and reconstructive surgery (see Table 4, 5, 6, and 7).

**Table 2:** An overview of near-infrared fluorescence imaging systems

NIRF system	Commercially available	FDA approval	System description	Fluorescence capability	No. of studies^a^	References
PDE	Yes	2012	Yes	820 nm	13	[7–19]
SPY	Yes	2005	Yes	650 nm 805 nm	16	[20–35]
FLARE	No	No	Yes	820 nm	8	[36–43]
Visionsense	Yes	2013	Yes	805 nm	1	[44]
Fluobeam	Yes	2014	Yes	750 nm	2	[45, 46]
LEICA	Yes	2015	Yes	635 nm 820 nm	2	[47, 48]
HyperEye	Yes	No	Yes	760 nm 780 nm	1	[49]
Pentero	Yes	2010	Yes	560 nm 635 nm 820 nm	1	[50]

*NIRF* near-infrared fluorescence, *FDA* Food and Drug Administration, *PDE* photodynamic eye, Photonics K.K. (Hamamatsu, Japan), *Visionsense* Visionsense ICG-NIR-VA system (Orangeburg, New York), *FLARE* fluorescence-assisted resection and exploration imaging system (Beth Israel Deaconess Medical Center, Boston), *LEICA* LEICA FL800, Leica Microsystems (Schweiz AG, Germany), *Pentero* OPMI Pentero IR800 (Carl Zeiss, Oberkochen Germany), *SPY* SPY elite, novadaq Technologies Inc. (Burnaby, British Columbia, Canada), *Fluobeam* Fluobeam Imaging Medical (Grenoble, France), *HyperEye* HyperEye Medical System (Tokyo, Japan)

^a^In four studies, no description was given of the imaging system

**Table 3:** An overview of NIR fluorescent dyes

Type	Dose^a^	FDA approval	Wave-length	Administration	Excretion site	No. of studies	References
ICG	0.1–5 ml 0.025–2.5 mg/kg 0.025 –25 mg^a^	Yes	800 nm	Intravenous subcutaneous	Liver	44	[7–39, 41–51]
MB	2.0 mg/kg^a^	Yes	665–688 nm	Intravenous	Kidney	1	[40]
DiR	N/A	No^b^	N/A	Labeled fat cells	N/A	1	[52]
LS601	N/A	No^b^	500–650 nm	Intraneural	Liver	1	[53]
HITC-H	N/A	No^b^	725 nm	Intraneural	N/A	1	[54]
LS851-H	N/A	No^b^	N/A	N/A	N/A	1	[54]
ADS740WS-H	N/A	No^b^	N/A	N/A	N/A	1	[54]
IRDye 800CW-H	N/A	No^b^	N/A	N/A	N/A	1	[54]

*ICG* indocyanine green, *MB* methylene blue, *DiR* 1,1′-dioctadecyl-3,3,3′,3′-tetramethylindotricarbocyanine iodide, *HITC-H* 1,1′,3,3,3′,3′-hexamethylindotricarbocyanine, *N/A* not available, *nm* nanometer

^a^Dose is described in heterogeneous manner

^b^Only in preclinical setting

**Table 4:** Applications of NIRF imaging in plastic and reconstructive surgery: angiography and perfusion imaging

Year of publication and author [reference]		Animal/clinical	Number	Imaging system	Dye	Dose	Administration
Group I. Angiography/perfusion imaging							
Ia Flap							
2009	Holm [50]	Clinical	50	Pentero	ICG	0.5 mg/kg	Intravenous
2009	Matsui [41]	Animal	22	FLARE	ICG	N/A	Intravenous
2009	Newman [35]	Clinical	8	SPY	ICG	2.5 mg/ml	Intravenous
2010	Lee [43]	Clinical	6	FLARE	ICG	N/A	Intravenous
2010	Matsui [42]	Animal	12	FLARE	ICG	0.07 mg/kg	Intravenous
2010	Quilichini [15]	Clinical	4	PDE	ICG	0.5 mg/kg	Intravenous
2010	Komorowska [32]	Clinical	24	SPY	ICG	5 mg/ml	Intravenous
2013	Ashitate [40]	Animal	15	FLARE	MB	2.0 mg/kg	Intravenous
2013	Wu [29]	Clinical	14	SPY	ICG	3.3–3.5 ml	Intravenous
2014	Munabi [27]	Clinical	42	SPY	ICG	2.5 mg/ml	Intravenous
2014	Nagata [11]	Clinical	30	PDE	ICG	N/A	Intravenous
2015	Daram [20]	Clinical	3	SPY	ICG	N/A	intravenous
2015	Hayashi [10]	Clinical	1	PDE	ICG	N/A	Intravenous
2015	Nasser [24]	Animal	54	SPY	ICG	2.5 mg/ml	Intravenous
2015	Sugawara [48]	Clinical	40	LEICA	ICG	25 mg	Intravenous
2015	Vargas [36]	Animal	4	FLARE	ICG	1.3 mg	Intravenous
2015	Watson [51]	Animal	5	Prototype^a^	ICG	0.5 mg/kg	Intravenous
2016	Bigdeli [44]	Clinical	8	Visionsense	ICG	0.5 mg/kg	Intravenous
2016	Diep [22]	Clinical	114	SPY	ICG	N/A	Intravenous
2016	Hitier [45]	Clinical	20	Fluobeam	ICG	0.025 mg/kg	Intravenous
2016	Kuriyama [49]	Clinical	11	Hyper Eye	ICG	0.1 mg/kg	Intravenous
2016	Ludolph [21]	Clinical	35	SPY	ICG	10 mg	Intravenous
2016	Xu [8]	Animal	18	PDE	ICG	0.2 mg/kg	Intravenous
2016	Bertoni [34]	Clinical	28	SPY	ICG	2.5 mg/ml	Intravenous
2016	Xu [7]	Animal	30	PDE	ICG	0.2 mg/kg	Intravenous
2017	Hammer-Hansen [33]	Clinical	66	SPY	ICG	N/A	Intravenous
Ib Bone flap							
2012	Nguyen [37]	Animal	8	FLARE	ICG	1.25 mg	Intravenous
Ic Abdominal wall							
2013	Patel [28]	Clinical	17	SPY	ICG	2.5 mg/ml	Intravenous
2016	Wormer [23]	Clinical^b^	95	SPY	ICG	5 mg	Intravenous
Id Composite tissue allograft							
2012	Nguyen [38]	Animal	8	FLARE	ICG	1.25 mg	Intravenous
2013	Nguyen [39]	Animal	5	FLARE	ICG	1.3 mg	Intravenous
2015	Valerio [25]	Clinical	16	SPY	ICG	2.5 mg/ml	Intravenous

*FDA* Food and Drug Administration, *NIRF* near-infrared fluorescence, *PDE* photodynamic eye, Photonics K.K. (Hamamatsu, Japan), *Visionsense* Visionsense ICG-NIR-VA system (Orangeburg, New York), *FLARE* fluorescence-assisted resection and exploration imaging system (Beth Israel Deaconess Medical Center, Boston), *LEICA* LEICA FL800, Leica Microsystems (Schweiz AG, Germany), *Pentero* OPMI Pentero IR800 (Carl Zeiss, Oberkochen Germany), *SPY* SPY elite, novadaq Technologies Inc. (Burnaby, British Columbia, Canada), *Fluobeam* Fluobeam Imaging Medical (Grenoble, France), *HyperEye* HyperEye Medical System (Tokyo, Japan), *ICG* indocyanine green, *MB* methylene blue, *IB* isosulfan blue, *DiR* 1,1'-dioctadecyl-3,3,3′,3′-tetramethylindotricarbocyanine iodide, *HITC-H* 1,1′,3,3,3′,3′-hexamethylindotricarbocyanine, *N/A* not available

^a^Characteristics of prototype not further specified by authors

^b^Randomized clinical trial

**Table 5:** Applications of NIRF imaging in plastic and reconstructive surgery: lymphography

Year of publication and author [reference]		Animal/clinical	Number	Imaging system	Dye	Dose	Administration
Group II. Lymphography							
IIa Composite tissue allograft							
2012	Mundinger [13]	Animal	9	PDE	ICG	0.03 mg	Subcutaneous Four different sites (0.2 ml/3 cm^3^ skin)
2017	Miranda Garcés [46]	Clinical	23	Fluobeam	ICG	0.5 ml	Intradermally into the edges of all flaps
IIb Staging lymphedema							
2014	Yamamoto [12]	Clinical	15	PDE	ICG	0.03 mg	Subcutaneous Hand: 2nd web space
2016	Narushima [9]	Clinical	N/A	PDE	ICG	N/A	Subcutaneous Hand: 2nd web space + ulnar border PL level wrist Foot: 1st web space + lat border AT
IIc Perioperative planning lymphaticovenous anastomosis							
2012	Maegawa [14]	Clinical	102	PDE	ICG	N/A	Subcutaneous Affected limb: four web spaces
2013	Chang [19]	Clinical	65	PDE	ICG	0.01–0.02 ml	Intradermally into each finger/toe web space
2014	Liu [47]	Clinical	20	LEICA	ICG	0.03 mg	Subcutaneous Hand: 2nd and 3rd web space + medial and lateral volar hand Foot: 1st and 3rd web space + medial and lateral side Achilles tendon
2016	Chen [31]	Clinical	21	SPY	ICG	0.25 mg	Subcutaneous Hand: 2nd and 3rd web space Foot: 1st and 2nd web space
2016	Shih [30]	Clinical	5	SPY	ICG	0.2 ml	Subcutaneous Foot: 2nd web space Hand: 2nd web space
2017	Ogata [16]	Clinical	5	PDE	ICG	0.03 mg	Subcutaneous Foot: 1st web space

*FDA* Food and Drug Administration, *NIRF* near-infrared fluorescence, *PDE* photodynamic eye, Photonics K.K. (Hamamatsu, Japan), *Visionsense* Visionsense ICG-NIR-VA system (Orangeburg, New York), *FLARE* fluorescence-assisted resection and exploration imaging system (Beth Israel Deaconess Medical Center, Boston), *LEICA* LEICA FL800, Leica Microsystems (Schweiz AG, Germany), *Pentero* OPMI Pentero IR800 (Carl Zeiss, Oberkochen Germany), *SPY* SPY elite, novadaq Technologies Inc. (Burnaby, British Columbia, Canada), *Fluobeam* Fluobeam Imaging Medical (Grenoble, France), *HyperEye* HyperEye Medical System (Tokyo, Japan), *ICG* indocyanine green, *MB* methylene blue, *IB* isosulfan blue, *DiR* 1,1'-dioctadecyl-3,3,3′,3′-tetramethylindotricarbocyanine iodide, *HITC-H* 1,1′,3,3,3′,3′-hexamethylindotricarbocyanine, *N/A* not available

**Table 6:** Applications of NIRF imaging in plastic and reconstructive surgery: neurography

Year of publication and author [reference]		Animal/clinical	N	Imaging system	Dye	Dose	Administration
Group III. Neurography							
2012	Gustafson [53]	Animal	3	N/A	LS601	N/A	Intraneural, sciatic nerve
2015	Tanaka [18]	Clinical	8	PDE	ICG	0.1 mg/kg	Intravenous
2016	Zhou [54]	Animal	24	N/A	4 new^a^	N/A	Intraneural

*FDA* Food and Drug Administration, *NIRF* near-infrared fluorescence, *PDE* photodynamic eye, Photonics K.K. (Hamamatsu, Japan), *Visionsense* Visionsense ICG-NIR-VA system (Orangeburg, New York), *FLARE* fluorescence-assisted resection and exploration imaging system (Beth Israel Deaconess Medical Center, Boston), *LEICA* LEICA FL800, Leica Microsystems (Schweiz AG, Germany), *Pentero* OPMI Pentero IR800 (Carl Zeiss, Oberkochen Germany), *SPY* SPY elite, novadaq Technologies Inc. (Burnaby, British Columbia, Canada), *Fluobeam* Fluobeam Imaging Medical (Grenoble, France), *HyperEye* HyperEye Medical System (Tokyo, Japan), *ICG* indocyanine green, *MB* methylene blue, *IB* isosulfan blue, *DiR* 1,1'-dioctadecyl-3,3,3′,3′-tetramethylindotricarbocyanine iodide, *HITC-H* 1,1′,3,3,3′,3′-hexamethylindotricarbocyanine, *N/A* not available

^a^Four new dyes: 1,1′,3,3,3′,3′-hexamethylindotricarbocyanine (HITC-H), LS851-H, ADS740WS-H, IRDye800CW-H

**Table 7:** Applications of NIRF imaging in plastic and reconstructive surgery: miscellaneous

Year of publication and author [reference]		Animal/clinical	Number	Imaging	Dye	Dose	Administration
Group IV Miscellaneous							
IVa Revascularization							
2014	Brooks [26]	Clinical	6	SPY	ICG	2.5 mg/ml	Intravenous
IVb Autologous fat grafting							
2015	Bliley [52]	Animal	24	N/A	DiR	N/A	N/A
IVc Trauma							
2016	Koshimune [17]	Clinical	23	PDE	ICG	0.2 mg/kg	Intravenous

*FDA* Food and Drug Administration, *NIRF* near-infrared fluorescence, *PDE* photodynamic eye, Photonics K.K. (Hamamatsu, Japan), *Visionsense* Visionsense ICG-NIR-VA system (Orangeburg, New York), *FLARE* fluorescence-assisted resection and exploration imaging system (Beth Israel Deaconess Medical Center, Boston), *LEICA* LEICA FL800, Leica Microsystems (Schweiz AG, Germany), *Pentero* OPMI Pentero IR800 (Carl Zeiss, Oberkochen Germany), *SPY* SPY elite, novadaq Technologies Inc. (Burnaby, British Columbia, Canada), *Fluobeam* Fluobeam Imaging Medical (Grenoble, France), *HyperEye* HyperEye Medical System (Tokyo, Japan), *ICG* indocyanine green, *MB* methylene blue, *IB* isosulfan blue, *DiR* 1,1'-dioctadecyl-3,3,3′,3′-tetramethylindotricarbocyanine iodide, *HITC-H* 1,1′,3,3,3′,3′-hexamethylindotricarbocyanine, *N/A* not available

### NIRF imaging systems

Various NIRF imaging systems have been described in the literature, as summarized in Table 2. In the described experiments, hand-held imaging systems and microscopes with an integrated NIRF were equally divided for imaging. There were four different hand-held systems (PDE *n* = 13, Visionsense *n* = 1, Fluobeam *n* = 2, and HyperEye *n* = 1) [7–19, 44–46, 49], one non-hand-held system (FLARE *n* = 8) [36–43], and three types of microscopes with an integrated NIRF (SPY *n* = 16, LEICA *n* = 2, and Pentero *n* = 1) [20–35, 47, 48] suitable for fluorescence image guidance. In one study, a prototype was used which was not further specified [51]; three articles unfortunately did not state what kind of imaging system was used [52–54].

### NIR fluorescent dyes

A handful of NIR fluorophores are reported in the literature (see Table 3). Indocyanine green (ICG) and methylene blue (MB) are two clinical fluorophores. Preclinical dyes have also been under investigation. Currently, the maximum penetration depth of NIRF visualization of ICG or MB is limited to 1.0–1.5 cm. The use of ICG was described in 44 articles [7–39, 41–51], thereby making it by far the most commonly administered dye. ICG was injected either subcutaneously in order to visualize superficial lymphatic vessels (i.e. lymphography) or intravenously in order to assess flap, composite allograft, or bone perfusion (i.e., angiography).

One study used intravenous MB to assess flap perfusion [40]. Although only once reported in plastic surgery literature, methylene blue is in fact a potential dye for near-infrared fluorescence imaging at around 700 nm.

Five different preclinical dyes were tested in animal studies in order to detect nerve injury by intraneural injection of the dye [53, 54]. One study labeled fat cells with a specific fluorescent dye to enable the investigation of the amount of fat cells, which survived after autologous fat cell transportation [52].

No side effects due to the administered dye were reported in the included studies. Nevertheless, although rare, the reported rates of severe and moderate reactions to ICG are approximately 0.07–0.1%. Additionally, methylene blue is also known to potentially cause severe allergic reactions as well.

### Applications of NIRF imaging in plastic and reconstructive surgery

NIRF imaging has already been explored for multiple applications in plastic surgery, either in an animal study or in a clinical setting. An overview of applications for tissue navigation is displayed in Table 4, 5, 6, and 7. Undoubtedly, angiography and lymphography are currently the two most used NIRF applications in plastic surgery.

NIRF angiography, after intravenous dye administration, was reported in 32 articles. The majority (*n* = 24) [7, 8, 10, 11, 15, 20–22, 24, 27, 29, 32, 35, 36, 40–44, 48–51] used NIRF to assess tissue perfusion in (free) flap surgery; the remainder focused on the perioperative assessment of mastectomy skin flap perfusion [32–34], bone perfusion [37], abdominal wall perfusion in abdominal wall reconstruction [23, 28], and perfusion of a composite allograft [25, 38, 39]. See Table 4. When reported, intravenous ICG dosage for perfusion imaging ranged from 0.025 to 0.50 mg/kg.

Ten articles [9, 12–14, 16, 19, 30, 31, 46, 47] used NIRF lymphography after subcutaneous/intradermal administration for a variety of reasons: to plan a lymphaticovenous anastomosis (LVA), to stage lymphedema, or to assess lymphatic flow in a composite allograft (e.g., vascularized lymph node transplants). See Table 5. When reported, the ICG dosage for lymphography ranged from 0.03 to 0.25 mg, which was administered subcutaneously/intradermally.

Three articles [18, 53, 54] injected a preclinical dye intraneurally to check for nerve injury (see Table 6).

There are some other novel applications within the field of plastic surgery (see Table 7). Dye administration in autologous transplanted fat tissue, for example, was investigated to assess the amount of fat cells that survived [52]. Another article [26] used NIRF to assess perfusion after revascularization of upper limb extremity ischemia. The application of NIRF imaging to determine tissue necrosis in open lower-limb fractures was also reported [17].

Unfortunately, the dosage and timing of administration of the different types of dye for the variety of aforementioned applications is either poorly documented and/or no consensus is available. A worldwide-accepted protocol for general clinical use is lacking. This would be of particular interest for the already clinically available dyes and applications.

## Discussion

The aim of this review was to evaluate the current applications (including available imaging systems and fluorescent dyes) and potential future applications of NIRF imaging in plastic and reconstructive surgery. NIRF imaging has shown potential for identification of several vital anatomical structures (e.g., arteries, veins, lymph vessels), even when covered under a layer of adipose or connective tissue. NIRF imaging can visualize vessels up to 1.5 cm subcutaneously [55]. These are all hollow structures that can be delineated using endo-luminal transported agents. Nerves have also been illuminated by intraneural injection and a dye, which is hydrophilic. However, future fluorescent dyes have been reported that will allow for solid anatomical structures to be visualized through NIRF imaging using specific peptides as targets [53, 56, 57]. The latter underlines the value for including animal studies within the current review. Preclinical fluorescent dyes have to be evaluated in animal setting first prior to human testing and validation. Inclusion of both animal and clinical studies is valuable to forecast future perspectives.

The current study comprises the first review in which all aspects (imaging systems, dyes, and clinical applications) of NIRF imaging within plastic and reconstructive surgery is discussed. Previous reviews only focused on one specific type of dye (ICG) or mainly one type of application. For example, Burnier et al. published a review on ICG applications in plastic surgery. Approximately half of their included studies reports on guidance during sentinel lymph node biopsy [58]. Liu et al. published a review on perioperative ICG angiography [59]. In both reviews, only ICG is used as NIRF dye.

From the systematic literature search, it can be concluded that NIRF is mainly used for angiography (e.g., flap perfusion) and lymphography (e.g., for perioperative planning of LVA and staging of lymphedema). Only a minority has described the potential for neurography using NIRF. However, in plastic and reconstructive surgery, enhanced nerve detection would also be of particular interest, for example in detecting or excluding nerve injury (i.e., differentiating between nerve injury versus neuropraxia), in the treatment of traumatic amputation of digit(s), guiding sensory free flap surgery, or facial nerve surgery.

Besides the aforementioned studies, publications on novel applications of NIRF image guidance in plastic surgery are scarce. Bliley et al. describe an in vivo technique in which stromal vascular fraction within autologous fat grafts can be tracked by NIRF [52]. This technique offers potential to determine the prevalence and destiny of injected fat cells in the future, thereby giving it a role in autologous fat grafts in reconstructive surgery, a surgical procedure which is being increasingly implemented in daily clinical practice and may become the future for the reconstruction of defects.

In case of a trauma, NIRF could be a convenient tool to determine soft tissue injury and necrosis thereby guiding trauma debridement. Koshimune et al. used NIRF to designate necrosis and reduce the number of debridement after open lower-limb fractures [17]. A precise assessment of skin defect size and the presence or absence of necrotic tissue can be useful in an estimation of flap size. Brooks et al. used NIRF to assess perfusion after revascularization of upper limb extremity ischemia. NIRF was used to increase understanding of the physiology of arterial-venous reversal in patients with terminal ischemia of an upper limb [26].

At the moment, ICG is the most frequently used dye in NIRF in plastic surgery. One of the advantages of ICG for NIRF angiography in particular is the quick half-life of 3–4 min in healthy adults. Therefore, it can be used several times for imaging without exceeding the maximal dosage [60]. In the available literature, dosage of different types of dye is either poorly documented or no consensus is present. Time between injecting and NIRF imaging, as well as distance of the camera to the target-tissue, is not unanimously defined. Currently, there is no standard protocol on dosage and timing of dye administration for general use.

Furthermore, no consensus is available on subcutaneous injection of ICG to visualize lymphatic vessels. In perioperative planning of LVA and staging lymphedema, ICG is injected subcutaneously in one or more of the web spaces of the foot or the hand depending on the location of the lymphedema. Additional injections are also given subcutaneously at the medial and volar side of the hand or at the medial and lateral side of the Achilles tendon. No agreement has yet been reached about which web space should be used, and whether additional injections are in fact necessary.

This review presented some limitations. The level of evidence of the included studies is rather low. Only one randomized clinical trial on abdominal wall perfusion could be included [23]. The majority of the studies were case reports, cohort studies, or (pre)clinical feasibility studies without a clear protocol regarding dosage, time of imaging, and administration. From this regard, a meta-analysis of available data could not be performed. Nevertheless, this study gives a comprehensive overview of the use of NIRF in the field of plastic surgery.

Further trials are needed to establish consensus regarding standard protocols for angiography and lymphography, two applications which are currently most applied within plastic and reconstructive surgery. This could be achieved by conducting (large, multicenter) randomized controlled trials. Next, other NIRF applications within plastic surgery need to be explored more extensively, such as NIRF-guided trauma debridement. Moreover, the imaging technique itself needs to be improved: more potent and powerful dyes would increase the range of applications as well as the penetration depth in tissues.

## Conclusion

Future standard implementation of novel intraoperative optical techniques, such as NIRF imaging, could significantly contribute to perioperative anatomy guidance and facilitate critical decision-making in plastic surgical procedures. Further investigation (i.e., large multicenter randomized controlled trials) is mandatory to establish the true value of this innovative surgical imaging technique in standard clinical practice and to aid in forming consensus on protocols for general use.
